# The Dual Role of Digital Self-Efficacy in Reading Engagement from a Nonlinear Dynamics Perspective

**DOI:** 10.3390/children12030292

**Published:** 2025-02-26

**Authors:** Mohammed Alghamdi, Georgios Sideridis

**Affiliations:** 1 Department of Self Development Skills, King Saud University, Riyadh 11451, Saudi Arabia; mhalghamdi@ksu.edu.sa; 2 Boston Children’s Hospital, Harvard Medical School, Boston, MA 02115, USA

**Keywords:** cusp model, bifurcation, reading engagement, digital self-efficacy, PIRLS 2021

## Abstract

**Background:** The purpose of this study is to elucidate the potentially complex relationship between digital self-efficacy and students’ engagement with reading activities, given that the students are known to enjoy reading. Of particular interest were the roles of digital self-efficacy as potential regulators and/or moderators of students’ engagement with reading activities at school. **Methods:** The participants were fourth-grade students from the Kingdom of Saudi Arabia. The data came from the Progress in International Reading Literacy Study (PIRLS) in 2021. The main hypothesis put forth was that digital self-efficacy would play a significant role in regulating students’ engagement with reading activities. The prediction of students’ reading engagement at school may take two different directions: linear and nonlinear. Based on the linear model’s prediction, it was expected that increases in digital self-efficacy would be associated with increases in engagement with reading activities. **Results:** The opposite prediction was supported; that is, heightened self-efficacy led to an unexpected drop and unpredictability in students’ engagement with reading. **Conclusions:** A potential explanation is that enhanced self-efficacy in digital skills is associated with enhanced interest and activity in digitally related tasks, such as video games, and engagement with internet-type activities and social media platforms, which consume a significant amount of time. Another possible explanation is related to levels of overconfidence in students, and it is suggested that those enhanced ratings are not necessarily associated with enhanced outcomes. It is concluded that enhanced digital self-efficacy may not have the expected positive effects as individuals become complacent and cease their efforts after seeing prior successes in reading achievement.

## 1. Introduction

This study evaluates the role of digital self-efficacy in understanding students’ reading engagement practices in the new age of information technology. Digital self-efficacy is defined as an individual’s perceived belief in their ability to perform tasks related to digital technologies. Such tasks may include adapting new technologies to learning, searching for information online, connecting with others via social media and online platforms, engaging in augmented reality, and communicating and collaborating via electronic means [1,2,3]. This new and evolving era, termed the digital or information age, involves the use of revolutionary electronic means that enhance access to information, make communication and connectedness easier, and improve productivity via automation and digitization [4]. It represents a rapidly evolving world in which it is deemed crucially important for individuals to adapt to new technologies so that they will develop critical personal and professional skills and competencies. Particularly in elementary schools, digital means are used to assist teaching and learning, and students need to be properly equipped to adapt their engagement to the requirements of the digital era. Given the strong relationship between students’ sense of self-efficacy and achievement [5,6,7,8,9], this study aims to evaluate the role of digital self-efficacy in students’ engagement with reading; it assumes an existing interest in reading and considers views asserting that the relationship may not be linear [10,11].

### 1.1. Reading Engagement and Liking of Reading

Recently, there has been a growing interest among scholars in understanding the relationship between the pleasure derived from reading and the level of active involvement in this intellectual pursuit. The academic community has been diligently exploring how the enjoyment of reading and the extent of engagement with the reading process are interconnected. The study in [12] provides an exploration of how reading motivation and engagement are interrelated, shedding light on their connection in both academic discussions and evaluation methods. Their work emphasizes not defining but quantifying the concepts of reading motivation and engagement, which are closely tied to an individual’s enjoyment of reading. In [13], the authors argue that people who actively participate in reading are more likely to be successful. This observation underscores the importance of involvement in achieving results, although it is also true that reading enjoyment causes a positive cascade of success.

### 1.2. Reading Engagement and Reading Self-Efficacy

Recent research has focused on the connection between individuals’ beliefs in the efficacy of their reading abilities and how engaged they are in the reading process. Several studies have explored this relationship, providing insights into how self-efficacy influences reading participation. In [14,15,16], the authors found a link between self-efficacy and people’s levels of engagement with reading activities. Building upon this line of inquiry, Ref. [17] investigated how beliefs about reading interact with an individual’s mindset regarding reading in order to predict reading goals and levels of engagement [18]. Their findings revealed that having a growth mindset rather than a fixed mindset is positively associated with levels of engagement. In the same vein, Ref. [15] examined the impact of self-efficacy in using digital reading tools on gender disparities and the interest in these tools. They discovered that lower levels of self-efficacy and interest contributed to better performance among female readers. Several studies have confirmed the positive relationship between reading efficacy and reading engagement and achievement [19,20,21,22], even though they used linear means [5,23]. This body of research helps us gain an understanding of how self-efficacy in reading relates to engagement with reading. However, one area that has received attention is the role of digital self-efficacy—which refers to an individual’s perceived beliefs about using technology to support their reading tasks—in shaping engagement with reading. This is because having skills helps individuals navigate and understand digital texts, resulting in a more active and fulfilling reading experience [24]. Given that digital technology is consistently adapted in the classroom, it is essential to evaluate how students engage with digital means. The movement from print to digital text has not been gradual. Studies have shown that students read much more digital text than they do printed text. Over 65% of 15-year-olds in the Organization for Economic Co-operation and Development (OECD) countries used digital texts for school-related reading once a week or more, and 35% preferred digital reading materials to print texts, according to the data collected by the Programme for International Student Assessment (PISA). In a similar vein, the Progress in International Reading Literacy Study (PIRLS) 21 reported that, in multiple educational contexts, engagement with digital reading was positively associated with students’ reading behaviors, comprehension strategies, and overall literacy outcomes [25]. Furthermore, research findings emphasize that students’ engagement with digital texts is mediated by their self-efficacy when using the digital platform, which affects their preference for digital reading tasks [26,27]. Therefore, the shift towards digital reading requires analysis, particularly of the impact of digital self-efficacy, which is recognized as an important predictor of reading engagement—especially because students with high levels of digital literacy skills tend to show different patterns of engagement from students with low and moderate digital competencies [28]. Failure to appreciate these dynamics may result in educational interventions that ignore the crucial aspects shaping students’ reading practices and academic success in a digitized learning context.

Examples of digital means include online platforms [29], the use of virtual classrooms by teachers, online cooperative activities [30], the contribution of information to class wikis or blog writing [31], the use of augmented reality experiments [32], the inclusion of infographic tools for informative presentations, the development of robotic activities, and personalized assessment strategies using e-portfolios and digital journals, to name a few [33,34]. Efficacious students are more likely to successfully interact with digital means and enhance their academic performance [26,35]. Given a digital era that points to a shift from print to digital formats, understanding how digital skills influence reading habits and comprehension strategies in modern education is crucial. As there is limited evidence on the role of digital self-efficacy, the question arises as to whether digital self-efficacy acts as a force that enhances student engagement or whether it becomes a hindering factor when levels of self-efficacy are low.

### 1.3. Proposed Analytical Model to Evaluate Hypothesized Relations

Reading is one of the most important skills for student achievement and school success. Reading skills are essential for extracting knowledge from language and non-language subjects; they are positive correlates of fluency and communication and thus have positive effects on both school success and career advancement. One of the most important correlates of reading skills is students’ engagement with reading. Research highlights the impact of reading enjoyment on student engagement and satisfaction [36]. Reading pleasure has been shown to improve pupils’ reading ability and accomplishment across populations and ages. The study in [37] found a strong link between reading engagement and joy in a meta-analysis of 52 empirical studies [r = 0.74]. Experimental studies have also supported the link between reading engagement and reading achievement [38]. Furthermore, Ref. [37] reported that students who were more engaged, particularly in terms of choosing and exercising autonomy in their reading activities, enjoyed reading more. This enjoyable experience creates a positive feedback loop that boosts participation [39] and subsequent achievement. One important goal of the study is to understand and predict reading engagement in students across languages and geographical areas.

**Figure 1:**
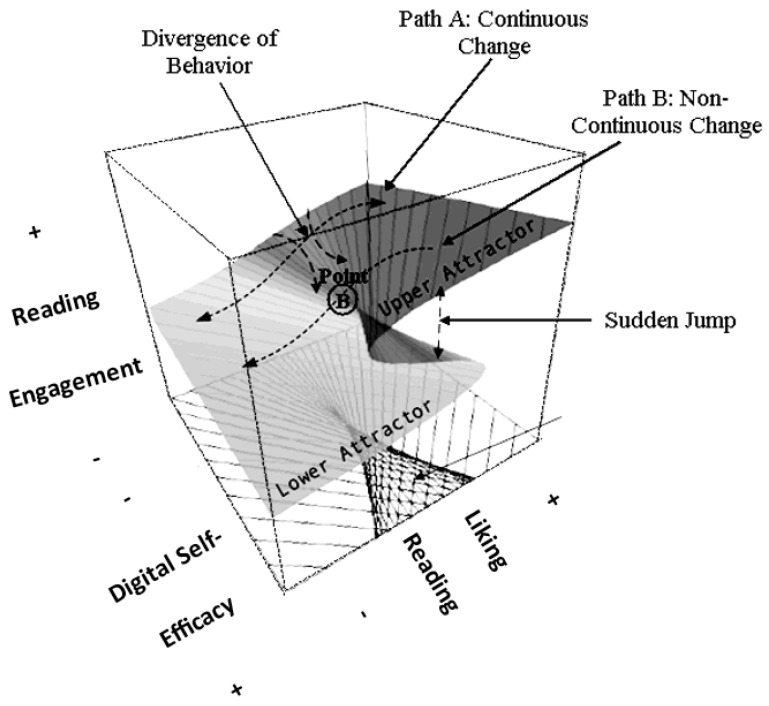
Description of the cusp model within the context of students’ engagement with reading activities (outcome variable) predicted by the linear effects of students’ liking of reading (asymmetry term) and the nonlinear effects of digital self-efficacy (bifurcation term).

In addition to reading engagement, a second important correlate of students’ achievement in reading is related to their feelings regarding reading-specific self-efficacy. Understanding the specific role and functioning of digital self-efficacy in influencing and regulating reading engagement is the primary goal of this study, which is important in at least two areas: the (a) societal and (b) scientific areas. Regarding (a), being technologically informed will likely contribute to societal goals, as economic growth and productivity are technology-driven. In Saudi Arabia, the most important challenges aligned with Vision 2030 are to attract global technology and adapt it to enhance efficiency in the public sector, modernize healthcare using technology, and reform the educational system. Regarding (b), we believe that the relationship between the importance and functioning of digital self-efficacy and reading engagement is complex and cannot be understood using linear analytical means. Instead, we hypothesize that this complex relationship can only be captured using nonlinear means, such as the cusp catastrophe model. Support for nonlinear analytical models was provided in the past by moderated mediational models [40] or U-shaped relationships [10]. In particular, the latter findings supported a quadratic relationship, and the authors of [10] concluded that school use of information and communication technologies (ICTs) should not interfere with students’ engagement with and learning of school subjects, as increases in the use of ICTs beyond a certain point were associated with decrements in achievement. The authors of [10] related the nonlinearity to automaticity effects, differential skill levels, and task difficulty, as individuals with more skills related to dealing with online tasks may access reading material more quickly compared to individuals with lower levels in the use of ICT [11,23,41,42,43]. Thus, the purpose of this study is to elucidate the potentially complex relationship between digital self-efficacy and students’ engagement with reading activities, given the fact that they enjoy and like reading. As briefly mentioned above, an important innovation of this study is the examination of the role of digital self-efficacy using the cusp catastrophe model. As shown in the figure below, the expectation is that, as digital self-efficacy increases, so will the joy and liking of reading and subsequent reading engagement. This relational path is depicted in “Pattern A to Linearity” in Figure 1. However, it is also predicted that, when digital self-efficacy levels become critically low, students may choose to disengage from reading activities because they do not have the necessary e-skills required to learn and perform reading tasks at school (such as when teachers use technology in their teaching). Thus, engagement with reading is expected to reach chaotic and unexpected levels when student levels of digital self-efficacy are below a critically low efficacy threshold. This relationship is depicted in “Pattern B to Nonlinearity”. Given the importance of digital self-efficacy in personal and societal development, it is of paramount importance to investigate its role and functioning in reading engagement.

### 1.4. Importance and Goals of This Study

There are several novelties of this study related to examining the role and functioning of digital self-efficacy in regulating student interest and engagement with reading activities. According to Bandura’s social cognitive theory, the beliefs that we have about our abilities significantly affect how we think, feel, and make choices [1,2]. When someone feels efficacious, it can greatly impact their reading abilities for various reasons. Firstly, in today’s world, where digital literacy is closely connected to reading skills, students who lack belief in their ability to use the tools may struggle to navigate and understand texts. This can hinder their progress in reading [27] and is especially concerning considering the increasing use of texts in education. Secondly, having strong efficacious beliefs in using technology may cause students to avoid opportunities for learning. As a result, they miss out on exposure to reading materials and interactive experiences that are important for developing reading skills [28]. Additionally, according to Bandura’s theory of motivation, students with efficacious beliefs are less likely to engage in self-regulated learning behaviors that are crucial for achieving success in reading. These behaviors include setting goals, monitoring progress, and seeking help when faced with difficulties while using texts [44]. In short, having high self-efficacy in utilizing technologies can significantly and adversely affect reading achievements by restricting access to online educational materials, decreasing interest in digital texts, and hindering the acquisition of self-directed learning behaviors that are crucial for successful reading in today’s digital era. This finding agrees with the study in [10], in which the authors advised caution against using ICT at school, as it may interfere with students’ learning of academic subject matters. Similar concerns were raised by the authors of [45], who proposed that the enhanced use of ICT may result in distractions and reductions in academic engagement [46]. Consequently, it is extremely important to evaluate the role of digital self-efficacy in reading engagement.

A second important novelty of this study is the idea stemming from past research that the relationship between self-efficacy and engagement may not be linear. In the past, most studies focused on the connection between self-confidence and reading ability; here, we present some evidence related to the strong positive relationship between self-efficacy and self-confidence in reading [47]. However, recent research has shown that this relationship is not always linear. For example, Ref. [48] highlighted how digital reading self-confidence may not follow a line when it comes to its impact on reading skills. In [49], the authors also pointed out that beliefs and achievements in children of all ages and skill levels may have a curvilinear relationship, suggesting that the previous studies focusing solely on connections may not have fully captured the complexity of this association. Moreover, Ref. [50] suggested that using questionnaires to measure self-efficacy beliefs could reveal stronger connections between reading self-ratings of efficacy and competence, pointing to a nonlinear pattern in the relationship. In this study, we use linear and nonlinear methodologies to unravel this complex relationship.

Thus, the purpose of this study is to elucidate the potentially complex relationship between digital self-efficacy and students’ engagement with reading activities, given that they enjoy reading. Of particular interest were the roles of digital self-efficacy as potential regulators and/or moderators of students’ engagement with reading activities at school; these roles are likely to be understood using nonlinear analytical means. This goal is particularly important given the advances in nonlinear modeling, such as the cusp catastrophe model, and the earlier nonlinear effects (e.g., quadratic) observed following the incorporation of ICT in the classroom.

## 2. Methodology

### 2.1. Participants and Procedures

The participants were 4th-grade students from the Kingdom of Saudi Arabia (girls = 2260, boys = 2126). To ensure student representativeness of their respective national populations, the PIRLS 2021 cohort utilizes a stratified two-stage cluster sampling design that is conducted as follows: In the first sampling stage, schools are sampled from among the entire list of schools in the target population that contain eligible students, using probability-proportional-to-size (PPS) sampling. These schools (or sampling frame) may be stratified (sorted) according to important demographic variables. At the same time, schools for the field test and data collection are sampled using a systematic random sampling approach. Each sampled school is replaced if it does not participate in the sample selection process according to a pre-assigned process using two replacement schools. Replacement schools are used only when the originally sampled schools do not participate. In the second sampling stage, one (or a few) class/classes is/are chosen from the target grade of each participating school. The coordinator for each country confirms that student participation rates are at least 80% among the available students in each country. The stratification procedure weighs the important factors, such as school type, geographic region, urbanization, and socioeconomic status. Furthermore, the calculation of weights is targeted at adjusting participant selection for school and student demographic characteristics. A series of post-stratification quality calibration procedures are in place to ensure the alignment of the samples with each national population. The procedures and methods implemented are described at [https://www.iea.nl/publications/technical-reports/methods-and-procedures-pirls-2021-technical-report, accessed on 24 February 2025].

### 2.2. Measures

All the measures were developed by the PIRLS Questionnaire Development Group (QDG), who have extensive experience in survey methodology and education policy analysis. The measures represent improvements over the measures administered by earlier cohorts. Instrument translation and adaptation underwent a rigorous review by linguistic experts [51]. The factorial validity and dimensionality of the measures were assessed using the Rasch partial credit model (PCM, [52]). The PCM relates the probability of a person selecting a specific response based on their position regarding the latent trait. The model assumes equal slope parameters at a fixed value of 1.0, and it assumes local independence and monotonicity, with the authors demonstrating alignment with assumptions [53].

#### 2.2.1. Reading Engagement

This construct was assessed using nine items and a 4-point scaling system ranging between “agree a lot” and “disagree a lot”. Sample items are “I like what I read about in school” and “My teacher gives me interesting things to read”. The unidimensionality of the scale was confirmed using the [52,54] partial credit model with discrimination parameters equal to one. The internal consistency reliability using Cronbach’s alpha was greater than 0.70. The raw cutoff scores were below 7.1 for little engagement, between 7.1 and 9.5 for medium engagement, and greater than 9.5 for high engagement.

#### 2.2.2. Digital Self-Efficacy

The scale comprises 8 items using a 4-point scaling system ranging between “agree a lot” and “disagree a lot”. Two sample items are “It is easy for me to find information on the internet” and “I know how to make and share a video”. The scale has undergone extensive study on its simple structure and internal consistency reliability, and both were adequate for the Saudi Arabia sample. For the simple structure in Ref. [52], the partial credit model was employed, given the polytomous nature of the data. The slope parameters were fixed to unity as per the tenets of the Rasch model. Participants with less than two scores on the scale were deleted from the measurements. The internal consistency reliability using Cronbach’s alpha was 0.78. For interpretation, the cutoff values for digital self-efficacy using the raw score metric were as follows: below 8.4, low efficacy; between 8.4 and 10.5, a medium level of efficacy; and above 10.5, a high level of efficacy.

#### 2.2.3. Liking Reading

Eight items are part of the “Students like reading” scale. They are rated using a Likert-type scaling system, with the options “agree a lot”, “agree a little”, “disagree a little”, and “disagree a lot”. Unidimensionality was verified using the partial credit model. Sample items are “I think reading is boring”, which is reverse-scaled, and “I learn a lot from reading”. The internal consistency using Cronbach’s alpha exceeded 0.70, which was adequate. The cutoff values using raw scores resulted in the following classifications: “do not like reading” when the raw scores were below 8.3; “somewhat like reading” when the raw scores were between 8.3 and 10.4; and “very much like reading” when the raw scores were greater than 10.4.

#### 2.2.4. Reading Achievement

Reading in the PIRLS involves a series of reading passages that are accompanied by a series of questions to evaluate comprehension and recall. The reading processes tested are (a) locating and recalling, (b) interpreting and integrating, (c) evaluating and critiquing, and (d) making connections. Several plausible values are created for validity purposes. These are correlated at more than 0.99 in most instances. Thus, in this study, we applied the first plausible value, although we tested the results with other plausible values to ensure the consistency of the findings.

### 2.3. Data Analyses

#### Cusp Catastrophe Model

The model known as the cusp catastrophe model, which was derived from catastrophe theory, provides a perspective on how systems undergo transformative changes as certain underlying variables gradually shift [55]. This model is characterized by a representation that vividly illustrates the changes in behavior that occur when specific predictor variables cross a threshold. Its applications span from neuropsychology and nursing research to behavioral studies, offering insights into abrupt shifts in nonlinear dynamic models. For instance, in psychology, the model has contributed to our understanding of how stress and coping mechanisms interact or impact one’s overall health. It explains how even slight increases in stress levels can suddenly trigger feelings of anxiety or depression [55]. The model in [56] sheds light on how anxiety and physiological arousal interact to shape performance, revealing a relationship that follows a “U-shaped” pattern with potential for changes. In ecology, the model has contributed to an understanding of how gradual environmental changes can lead to shifts within ecosystems [57]. Other applications have emerged in economics and geology. In the field of economics, this model provides insights into market crashes and financial crises by emphasizing the interplay between the economic factors that can lead to market collapse. Nevertheless, the model’s applicability is occasionally questioned due to the influence of individual and contextual factors on its predictions [55].

This model, predicated on the rate of change of a potential function U in relation to the outcome y, adeptly handles both linear and nonlinear relations through a higher-order probability density function. This surface hosts multiple equilibrium states whose existence and stability are heavily influenced by the interplay between these control components [58]. The cusp model is evaluated using the function of two control parameters, the asymmetry parameter “a” and the bifurcation term “b”, for the prediction of outcome variable “y”. The dynamics of this nonlinear system are captured by the potential function, as shown below:

V [y;α,β] = α y + 1/2 βy^2^ − 1/4 y^4^ (1)

As levels in the asymmetry and bifurcation variables vary, the model adjusts gradually or dramatically to these changes. The behavior of the system can change dramatically as the control parameters (a) and (b) vary, leading to the characteristic sudden jumps or “catastrophes” when the system moves between different equilibrium states. The equilibrium states are found by setting the derivative of the potential function with respect to [y] to zero, as shown below:

dV/dy = y^3^ − b_y_ − a = 0 (2)

All analyses were conducted using the cusp package in R 4.4.2 [59].

## 3. Results

### 3.1. Prerequisite Cusp Model Assumptions

**Figure 2:**
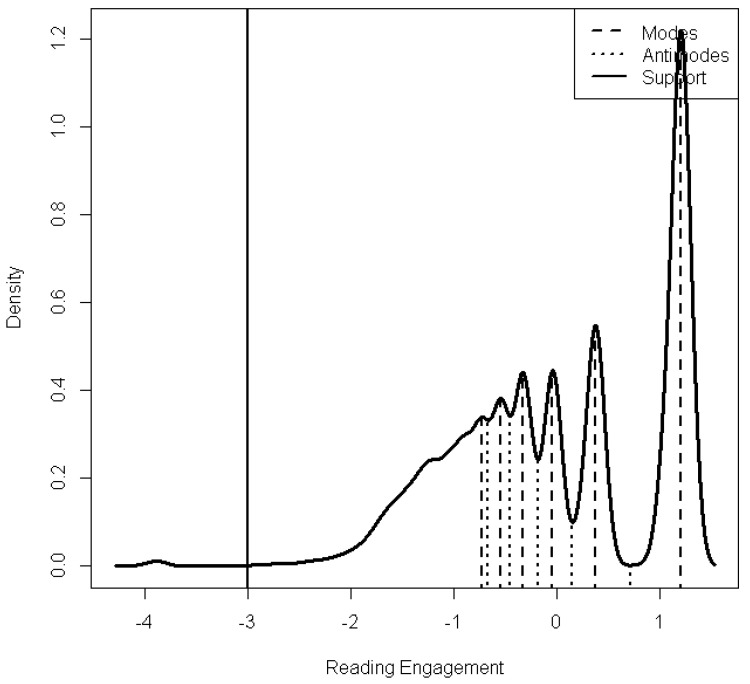
Multimodality in the measurement of reading engagement.

In terms of cusp model assumptions, the most important of all is the presence of bimodality or multimodality in the distribution of the outcome variable. The remaining tenets of the model were tested and are shown in a comparative form in Table 1 and Table 2. Multimodality was tested using the Multimode and Diptest packages in R. The inferential statistical test applied was from Hartigan and was significant [D = 0.974, *p* < 0.001]. The number of modes from the Gaussian kernel was estimated using the nmodes function in Multimode. The results revealed the presence of six distinct modes, thus supporting multimodality. Figure 2 displays the six modes inferred from the data.

### 3.2. Cusp Model Support

After satisfying the multimodality assumption, a full evaluation of the cusp catastrophe model, along with its competing linear model, was conducted. Table 1 displays the findings related to the functioning of the asymmetry and bifurcation variables and to the prediction of reading engagement. All the variables entered the model in standardized form [z-scored]. As shown in the table, all the intercept and slope tests were significantly different from zero. The role of the bifurcation terms is particularly important in reaching a conclusion favoring the cusp model over the competing models. Second, the cusp model was superior to the linear model using both information criteria (see Table 2), for which low values are preferred, and using the chi-square difference test for nested models [χ^2^ (2) = 5605.000, *p* < 0.001]. Thus, the inferential statistical means favored the cusp model over the linear model.

Of particular interest in the above results is the functioning of the bifurcation term, with implications for both the presence of a cusp and its directionality. A positive sign in the bifurcation term suggests that, at low levels of digital self-efficacy, its relationship with reading engagement is linear and positive. However, as levels of digital self-efficacy increase, reading engagement becomes unpredictable and chaotic. Under these lenses, gradual increases in self-efficacy are adaptive as expected, but high levels may lead to a phenomenon characterized by overconfidence in one’s abilities. Thus, although one would expect that low levels of digital self-efficacy would be particularly detrimental to reading engagement, this phenomenon was not observed; instead, a phenomenon of likely overestimation and overconfidence was observed, which, rather than leading to engagement, probably leads to withdrawal from reading activities. For the verification of digital self-efficacy functioning as a bifurcation parameter after controlling for various covariates (e.g., confidence in reading, home support, disruptive behaviors in the classroom, students’ feelings of belongingness in school, and the presence of bullying), the results are presented in the Supplementary Materials.

**Figure 3:**
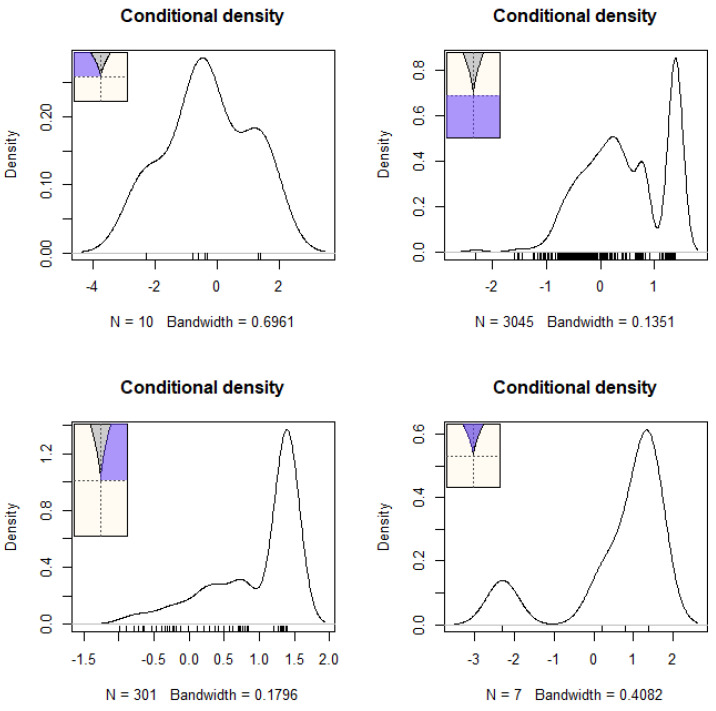
Densities of outcome variables across areas of the response surface.

**Figure 4:**
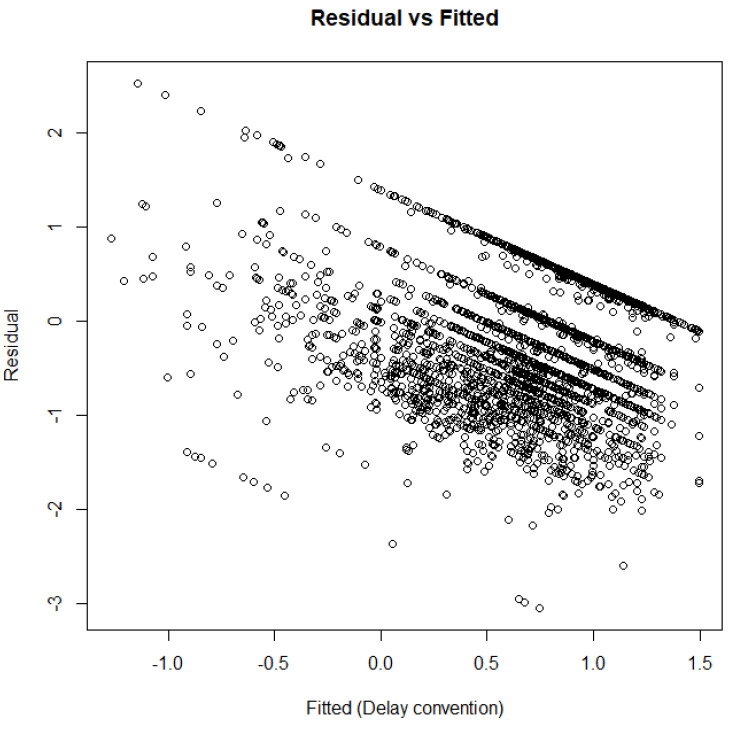
Residual vs. fitted estimates from the cusp model.

Further tenets of the cusp model are shown in Figure 3 and Figure 4. Figure 3 above displays the densities of the outcome variable across various areas of the response surface. The expectation is that data would be present across all areas and that bimodality or multimodality would also be evident in those distributions, especially closer to the bifurcation area (bottom right area). As shown in Figure 3, the observations spanned all quadrants of the response surface, with a substantial number being located within the bifurcation area (bottom right figure). Figure 4 displays a scatterplot with observed vs. fitted estimates, for which the expectations for a well-fitted model are (a) a zero relationship (or slightly negative, as shown in [59]) and (b) a limited spread. As shown in Figure 4, the expected negative pattern was observed, and the observations were closely related to each other, thus pointing to a good model fit.

**Figure 5:**
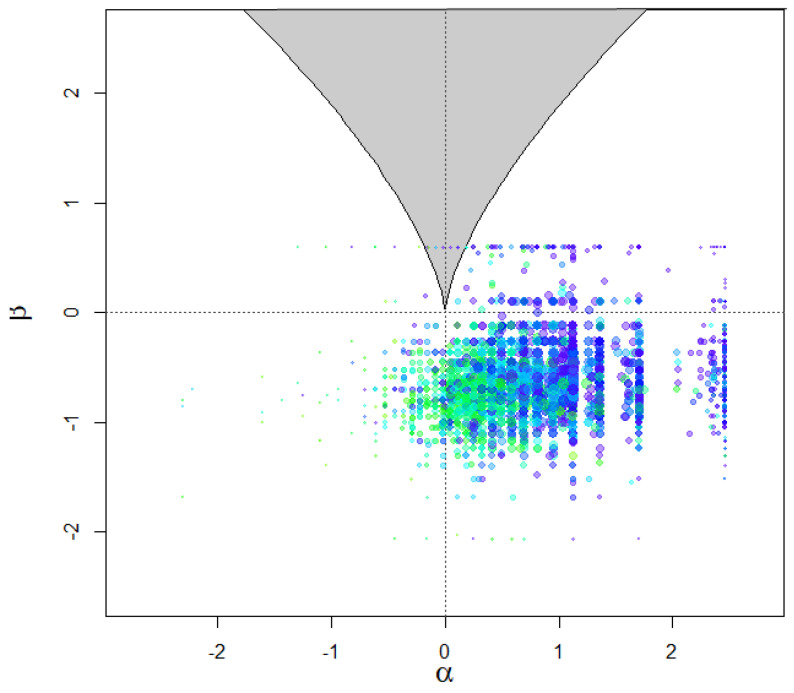
Relative location of observations in relation to distance between upper and lower surfaces. Darker colors are closer to the upper surface and lighter colors closer to the lower surface.

**Figure 6:**
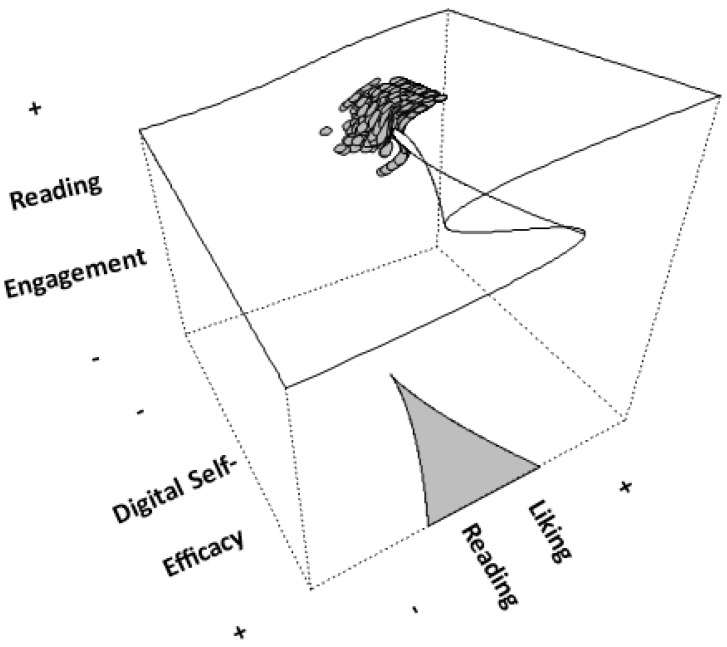
Three-dimensional figure displaying the observations and their placements within the cusp catastrophe model.

Figure 5 displays an important attribute of the cusp model, i.e., the transition of observations from upper to lower surfaces. Two figures are provided for that purpose: Figure 5 and Figure 6. Figure 5 shows the observations with different colors so that the reader can locate the observations across surfaces. The observations with darker colors are placed closer to the upper surface, and those with lighter colors are closer to the lower surface. As shown in the figure, the observations were of variable colors, suggesting the movement of participants between surfaces as the asymmetry and bifurcation terms took on various values.

## 4. Discussion

This study aimed to explore the connection between digital self-efficacy and students’ involvement in reading activities when they have a genuine interest in reading. We were particularly interested in understanding how digital self-efficacy could regulate students’ engagement with reading activities at school, especially in the Kingdom of Saudi Arabia, where levels of reading in fourth graders are well below the international average scoring and in the lower tier of the rankings (47th out of 57 countries) in relation to other countries.

A significant finding of this study is that, at low to medium levels of digital self-efficacy, engagement with reading is linear and positive. For example, a student who gradually becomes more confident in their abilities is expected to increase their engagement with a task as their ability grows and steadily improves. However, there may be a point where this increased confidence might lead to overconfidence [60]. This overconfidence could then result in a decline in performance because the student underestimates task difficulty or overestimates their preparedness [61]. This scenario showcases how surpassing a threshold of self-efficacy can trigger an unexpected behavior change [2]. This unexpected finding may indicate a bias where individuals with knowledge or competence tend to overestimate their capabilities [62,63]. Therefore, having high self-efficacy may result in a feeling of being in control and, consequently, in being bored with the task at hand; this may potentially lead to disengagement and a loss of focus, interest, and motivation [64]. Thus, a potential mechanism of overconfidence may be operative in that highly efficacious individuals may engage superficially with reading, may lose focus easily, or may take shortcuts (such as skimming rather than being deeply engaged in reading), leading to inconsistent engagement practices [65,66]. Another potential mechanism is cognitive overload. Highly competent and efficacious individuals may feel overwhelmed by the amount of information that they need to process because high levels of digital efficacy may lead to multitasking; individuals may shift their attention across various tasks without maintaining a sustained focus, which is required to deeply process reading information. This cognitive overload may then lead to disengagement from the reading task at hand, which partly explains the unpredictability of the engagement that results at high levels of digital self-efficacy.

### 4.1. Implications for Educational Policy

The findings on the connection between digital self-efficacy and student engagement with reading have significant implications for public policy. Given that low levels of digital self-efficacy are likely linked to low levels of engagement with reading activities, educational policies may target prioritizing digital literacy programs early on so that prerequisite skills (e.g., navigation on electronic platforms) are mastered and self-efficacy can be developed. Empowering teachers to also improve their skills and competencies in the use of digital tools will enhance their use as teaching tools but will first increase the teachers’ skills and competencies in engaging with such means. For example, teachers’ use of gamification, scaffolding, and digital collaboration [3,30] may be integrated into teaching [32]. Engaging more skilled students as tutors suggests the implementation of mentorship programs for that purpose.

### 4.2. Study Limitations and Future Directions

The cusp catastrophe model has some statistical shortcomings, even though it is well suited to the study of nonlinear dynamics in behavioral data. The complexity and sensitivity to the initial conditions of the model mean that estimating and interpreting parameters accurately may be a challenging task [67,68]. Large sample sizes are required to achieve sufficient power and reliability in complex models such as these, given that there may be circumstances in which finding a population large enough is not possible in educational research [69]. Additionally, the model’s reliance on continuous as opposed to discrete data makes it unusable for situations in which the original measurements are categorical [55]. Moreover, the cusp catastrophe model assumes that data are homoscedastic, meaning that the variance in the dependent variable is constant across levels or categories of the independent variable; however, in real-world datasets, this assumption may be violated [58]. Lastly, although we speculated that overconfidence may be a potential explanation for the bifurcation effects, research evidence suggests that overconfidence may be merely a statistical artifact [70].

## 5. Conclusions

In conclusion, this study’s investigation into the relationship between digital self-efficacy and student engagement with reading activities offers several new areas of information on how this relationship is manifested at the elementary school level. First, it is evident that the nonlinear model utilized here offers an advantage in terms of understanding the complex functioning of digital self-efficacy in that low levels are inconsistently linked to engagement. The fact that two different pathways of efficacy are potentially operative, leading to distinct reading engagement practices, is extremely valuable, as enhanced self-efficacy should not always be considered adaptive. To date, research has only pointed to the positive effects that result from feeling efficacious about academic tasks. The previously reported modest positive correlations may be due to the operation of the overconfidence effect. The fact that there is a risk that self-reported evaluations of one’s competencies may demonstrate overconfidence also has important implications for teaching and learning. Teachers’ use of self-regulation practices that involve self-correction or the correction of “others” by engaging pairs of students may be a highly effective way for students to validly evaluate their skills and competencies.

## Figures and Tables

**Table 1:** Parameter estimates of the cusp model for the prediction of reading engagement from liking reading and digital self-efficacy.

Cusp Model Coefficients	Slope	S.E.	*Z*-Test	*p*-Value
a_0_ [Intercept]	−3.084	0.132	−23.320	<0.001 ***
a_1_ [Liking Reading]	0.372	0.011	34.650	<0.001 ***
b_0_ [Intercept]	−2.723	0.139	−19.560	<0.001 ***
b_1_ [Digital Efficacy]	0.224	0.015	15.120	<0.001 ***
w_0_ [Intercept]	−3.111	0.044	−71.070	<0.001 ***
w_1_ [Reading Engagement]	0.338	0.003	97.700	<0.001 ***

Note: The intercept term of digital self-efficacy was deleted because its estimation error was out of bounds. The terms a, b, and w refer to asymmetry, bifurcation, and outcome variables’ intercept and slope terms, respectively. Intercepts are denoted by the “0” subscript and slopes by “1”. *** *p* < 0.001.

**Table 2:** Linear logistic and cusp model comparison.

Models Tested	Loglikelihood	Parameters	AIC	AICc	BIC
1. Linear	−6992.798	4	13,993.596	13,993.608	14,018.079
2. Logistic	−6950.741	5	13,911.482	13,911.500	13,942.085
3. Cusp	−4190.135	6	8392.269	8392.294	8428.993

## Data Availability

Data are available from the official study of PISA 2018 at [https://pirls2021.org/, accessed on 24 February 2025].

## Supplementary Materials

The following supporting information can be downloaded at: https://www.mdpi.com/article/10.3390/children12030292/s1, Table S1: Parameter Estimates of the Cusp Model for the Prediction of Reading Engagement from Liking Reading and Digital Self-Efficacy.
