# Supplementary material for: The Dual Role of Digital Self-Efficacy in Reading Engagement from a Nonlinear Dynamics Perspective
Source: Children (Basel). 2025 Feb 26;12(3):292. doi: 10.3390/children12030292 (PMC11941528; doi:10.3390/children12030292)
Supplement: Supplementary file 1 [file children-12-00292-s001.zip › children-3435580-supplementary.pdf]

Supplementary Material

To account for the potential confounding role of covariates, the cusp model was repeated with asymmetry variables (linear predictors) being confidence in reading, home support, disruptive behaviors in the classroom, students' feelings of belongingness to school, and the presence of bullying. Table S1 below shows the results from fitting the cusp-catastrophe model to this data.

Table S1. Parameter Estimates of the Cusp Model for the Prediction of Reading Engagement from Liking Reading and Digital Self-Efficacy.

Parameter Estimates	Estimate	Std. Error	z-test	p-value
a[(Intercept)]	-6.994	0.216	-32.344	<0.001***
a[likeread]	0.274	0.015	18.092	<0.001***
a[belong]	0.380	0.015	25.954	<0.001***
a[disord]	0.030	0.012	2.428	0.0152*
a[confident]	-0.012	0.013	-0.922	0.365
a[bull]	0.030	0.014	2.145	0.0319*
a[homesup]	0.167	0.031	5.364	<0.001***
b[(Intercept)]	-3.042	0.148	-20.559	<0.001***
b[se]	0.198	0.014	14.321	<0.001***
w[(Intercept)]	-3.207	0.045	-71.446	<0.001***
w[eng]	0.340	0.004	97.106	<0.001***

Note: likeread=liking reading, belong= students' feelings of belongingness to school, disord= disruptive behaviors in the classroom, confident=confidence in reading, bull=presence of bullying, homesup=number of home supports, se=digital self-efficacy, eng=reading engagement. * $p < 0.05$; *** $p < 0.001$.

As shown in the table, even after controlling for the presence of these covariates, the catastrophic relationship between digital self-efficacy and student engagement with reading activities remained strong and positive confirming the original hypotheses and the theses of the main study.
